# Impact of a Novel Hydrogel with Injectable Platelet-Rich Fibrin in Diabetic Wound Healing

**DOI:** 10.1155/2023/7532637

**Published:** 2023-07-28

**Authors:** Limin Bai, Xiaowei Zhang, Xiaomei Li, Susu Wang, Yeshun Zhang, Gang Xu

**Affiliations:** ^1^Department of Burn and Plastic Surgery, Northern Jiangsu People's Hospital, Yangzhou 225001, China; ^2^College of Biotechnology, Jiangsu University of Science and Technology, Zhenjiang Jiangsu 212100, China; ^3^Key Laboratory of Silkworm and Mulberry Genetic Improvement, Ministry of Agriculture and Rural Affairs, Sericultural Research Institute, Chinese Academy of Agricultural Sciences, Zhenjiang Jiangsu 212100, China; ^4^Clinical Medical College, Yangzhou University, Yangzhou 225009, China

## Abstract

Diabetic wounds are serious complications caused by diabetes mellitus (DM), which are further exacerbated by angiogenesis disorders and prolonged inflammation. Injectable platelet-rich fibrin (i-PRF) is rich in growth factors (GFs) and has been used for the repair and regeneration of diabetic wounds; however, direct application of i-PRF has certain disadvantages, including the instability of the bioactive molecules. Sericin hydrogel, fabricated by silkworm-derived sericin, is a biocompatible material that has anti-inflammatory and healing-promoting properties. Therefore, in this study, we developed a novel hydrogel (named sericin/i-PRF hydrogel) using a simple one-step activation method. The *in vitro* studies showed that the rapid injectability of the sericin/i-PRF hydrogel allows it to adapt to the irregular shape of the wounds. Additionally, sericin hydrogel could prolong the release of i-PRF-derived bioactive GFs in the sericin/i-PRF hydrogel. Furthermore, sericin/i-PRF hydrogel effectively repaired diabetic wounds, promoted angiogenesis, and reduced inflammation levels in the diabetic wounds of nude mice. These results demonstrate that the sericin/i-PRF hydrogel is a promising agent for diabetic wound healing.

## 1. Background

Chronic wounds caused by diabetes mellitus (DM) are a global public health concern and are expected to affect 439 million adults worldwide by 2030 [1]. Diabetic wounds are characterized by persistent inflammation, vascular compromise, bacterial infection, and inhibition of reepithelialization [2]. Moreover, a bacterial infection of diabetic wounds may induce the release of proinflammatory cytokines, such as interleukin- (IL-) 6 and tumor necrosis factor- (TNF-) *α*, resulting in delayed wound healing or nonhealing. Wound healing is divided into four phases, including the hemostatic, inflammatory, proliferative, and remodeling phases [3]. Many growth factors (GFs) and cells, such as platelets, macrophages, and fibroblasts, play key interdependent roles in the wound-healing process [4]. Numerous studies have shown that GFs play an important role in skin regeneration by influencing angiogenesis, granulation, and inflammatory processes [5]. However, DM-induced hyperglycemia may prevent wound healing by interfering with the wound-healing processes, angiogenesis, and immune regulation. In general, diabetic wounds are traditionally treated by wound dressing, surgical debridement, and wound repair by a skin graft or flap [6]. However, due to the impaired cellular function of diabetic wounds, such as insufficient production of bioactive molecules, many patients show poor prognoses with conventional treatment methods. Currently, GF therapy is a promising treatment for diabetic wounds; although, further studies are required to elucidate treatment methods to effectively and economically transport active molecules to the highly catabolic wound sites.

Platelet concentrates are products derived by centrifuging blood that has an above-normal concentration of platelets, and they are widely used in dentistry, orthopedics, and plastic surgery. Injectable platelet-rich fibrin (i-PRF) is prepared by using the low-speed centrifugation concept (LSCC) and contains uniformly distributed GFs. Furthermore, since only a small proportion of the platelets are activated, the liquid state of i-PRF can only be maintained for approximately 15 min [7]. However, i-PRF can transition from liquid to solid after the activation of thrombin, which allows fibrinogen in the platelets to cross-link to form fibrin. This phase transition property of i-PRF allows it to be used in combination with other biomaterials [8]. I-PRF contains platelets, GFs, leukocytes, and fibrin that can play important roles in different wound-healing phases [9]. When platelets are activated, *α*-granules can release a large number of bioactive factors, such as insulin-like growth factor, vascular endothelial growth factor (VEGF), platelet-derived growth factor (PDGF), fibroblast growth factor-basic, transforming growth factor- (TGF-) *β*, and other angiogenesis-related proteins [10, 11]. These GFs stimulate stem cell proliferation, angiogenesis, and epidermal growth by activating downstream signaling pathways, thereby promoting tissue repair and regeneration [12]. In addition, the fibrin reticular structure binds to the bioactive molecules before they attach to the corresponding cell surface receptors, allowing for their slow release as the fibrin matrix degrades [13]. The fibrin network also contains a large number of leukocytes, immune cells, and immunomodulatory factors, such as ILs and TNF, which are involved in immune cell recruitment and can directly induce platelet aggregation or indirectly induce chemotactic leukocytes to initiate and advance the healing process [14]. However, when used directly on diabetic wounds, the fibrin in i-PRF is easily decomposed by the enzymes at the wound site, further causing GF degradation, and thus hindering angiogenesis and wound healing [15]. Therefore, the synthesize of a hydrogel carriers that consumes proteolytic enzymes as a competitive protein and allows the continuous release of bioactive molecules in the wound is highly significant.

In general, hydrogel refers to a 3D hydrophilic and biocompatible polymeric mesh. The high water content of a hydrogel moisturizes the wound, facilitates cell migration, and promotes epithelialization. In addition, it can be used as a biological scaffold to protect the cells and their secreted bioactive substances [16]. An ideal hydrogel dressing for diabetic wounds should be porous and biocompatible, should have adjustable biodegradability, and should facilitate the continuous release of bioactive molecules [17, 18]. Silk protein is a new-generation natural polymer for wound healing that is biocompatible, permeable, and enzymatically degradable. Sericin is a large protein that is produced in the middle silk gland of the silkworm and accounts for approximately 20–30% of the cocoon weight. It consists of 18 amino acids, mainly composed of polar side chains, forming a complex of three main polypeptides, with serine being the main component of silk glue (approximately 30%). Sericin has good biocompatibility and low immunogenicity [19]. In addition, sericin exhibits several important biological properties, including antioxidant, moisturizing, anti-inflammatory, and regeneration properties [19, 20]. Studies have shown that sericin hydrogels are simple to prepare at low cost, are easy to store, and have a long shelf life. When applied to severe skin injuries, they may facilitate skin regeneration by inhibiting inflammation, stimulating VEGF and epidermal growth factor (EGF) expression, regulating TGF-*β*1 and TGF-*β*3 expression, and promoting stem cell migration to the injury site to increase cell proliferation [21]. Moreover, some studies have reported that sericin hydrogels can be used as carriers for controlled drug release [22].

In this study, we synthesized sericin/i-PRF hydrogel which provided a dense porous matrix for i-PRF, allowed uniform platelet distribution, and prolonged GF release. The biosafety of the sericin/i-PRF hydrogel was confirmed by cytotoxicity tests and *in vivo* degradation tests in nude mice. Application of the sericin/i-PRF hydrogel to diabetic wounds modulated inflammation and promoted angiogenesis and collagen deposition, thus significantly enhancing wound healing in nude mice.

## 2. Materials and Methods

### 2.1. Materials

Natural fibroin-deficient mutant silkworm (*Bombyx mori*) cocoons (185 Nd-s) were obtained from the Sericultural Research Institute, China Academy of Agriculture Science (Zhenjiang, Jiangsu, China). Cellulose dialysis membranes (3500 Da MWCO) were purchased from Biosharp Technology (Beijing, China). I-PRF was obtained from Northern Jiangsu People's Hospital (Jiangsu, China), while L929 mouse fibroblasts (L929s) were purchased from Xinyuan Technology (Guangdong, China). The MTT kits were purchased from Solarbio (Beijing, China), and enzyme-linked immunosorbent assay (ELISA) kits were purchased from Boster Biological Technology Co, Ltd (Wuhan, China). Lastly, the nude mice were purchased from Yangzhou University (Jiangsu, China).

### 2.2. Preparation of the Sericin/i-PRF Hydrogel

#### 2.2.1. Preparation of Sericin Solution

Sericin was isolated from natural fibroin-deficient mutant *B. mori* cocoons using the lithium bromide (LiBr) method. The cocoons were lysed in 6 M LiBr aqueous solution at 37°C, then dialyzed and concentrated to obtain the desired concentration of sericin solution.

The cellulose dialysis membranes were pretreated by boiling the membranes in 500 mL each of 2% (*w*/*v*) sodium bicarbonate solution and 1 mmol/L EDTA solution (pH 8.0) for 10 min, after which they were cooled and set aside until further use.

To isolate sericin, 600 mg of cocoon was added to a reagent bottle with 24 mL of 6 M LiBr solution and incubated at 36.5°C in a water bath for 24 h. The mixture was then centrifuged at 4000 rpm for 5 min to remove the insoluble material. Thereafter, 6 mL of Tris-HCl (1 M, pH 9.0) was added to the above solution and transferred to the cellulose dialysis membrane. The membrane was then placed in a reagent bottle containing ultrapure water, which was stirred slowly and replaced every 6 h. After 48 h of dialysis, the filamentous solution was concentrated with PEG 6000 aqueous solution. Thereafter, the solution was centrifuged at 4000 rpm for 5 min, and the precipitate was removed. The concentration of the sericin solution was determined by bicinchoninic acid assay or dry method. Lastly, sterile ddH_2_O was used to dilute the solution to 2% (*w*/*v*), and sericin hydrogel was prepared by mixing sericin solution and 25% (*w*/*v*) glutaraldehyde (Solarbio, China) in a 100 : 2 (*v*/*v*) ratio.

#### 2.2.2. Preparation of i-PRF

Venous blood (10 mL) was collected from the volunteers and placed in sterile plastic centrifuge tubes (Kangjian, China) and centrifuged (TD5P, Shanghai Lu Xiang Yi) at 700 rpm for 3 min at 25°C. Thereafter, approximately 1 mL of the yellow liquid (i-PRF) from the upper layer was obtained, and its concentration was determined. The concentration of the platelets in i-PRF was found to be 600 × 10^9^/L. This study was performed in accordance with the approved Medical Ethics Committee research protocol (Ethical approval number: 2022ky260).

#### 2.2.3. Preparation of the Sericin/i-PRF Hydrogel

Firstly, 1 mL of 2% aqueous sericin solution was mixed with 1 mL of i-PRF to form a homogeneous solution. Thereafter, 30 *μ*L of 25% glutaraldehyde solution was added to the above mixture. This mixture was incubated at 25°C, during which it underwent a liquid-to-solid transformation to form the sericin/i-PRF hydrogel.

### 2.3. Gel Characterization

#### 2.3.1. Microstructure Observation

Sericin hydrogel, i-PRF, and sericin/i-PRF hydrogel were frozen overnight at -80°C and transferred to a cryogenic vacuum desiccant to freeze dry. Thereafter, the samples were placed under a scanning electron microscope (SEM; Gemini SEM 300, UK) and observed at 10.0 kV.

#### 2.3.2. Gelation Time

The gelation times of the sericin hydrogel, i-PRF, and sericin/i-PRF hydrogel were determined by placing the samples (2 mL each) in a 5 mL bottle, then incubating the samples at 4, 25, and 37°C in a water bath and tilting the bottles every 30 s until solidification.

#### 2.3.3. Swelling Ratio

The swelling ratio of the gels was determined by the gravimetric method [23]. Briefly, the gels were weighed and then fully immersed in acidic, neutral, and basic phosphate buffer solutions (PBS; pH 3.0, 7.4, and 11.0, respectively) at 37°C. The gels were removed from the PBS at 2, 4, 6, 8, 12, 16, and 24 h; gently dried with filter paper; and immediately weighed (*W*_*t*_). The swelling ratios of the gels were determined using the following formula:

(1) $$\begin{array}{c} \text{Swelling ratio}\left(g/g\right)=\left(W_{t}-W_{0}\right)/W_{0}, \end{array}$$

where *W*_*t*_ is the weight of the gels at time *t* and *W*_0_ is the initial weight of the gels.

#### 2.3.4. Rheological Properties

The rheological properties of the gels were measured using a rheometer (DHR-2, TA Instrument, USA). All the samples were molded into 25 mm diameter discs. The time scan experiments were tested at a fixed frequency of 1 Hz at a strain of 1% at 25°C, while the frequency scan experiments were conducted at shear frequencies of 0.1 to 10 Hz. The energy storage modulus (G') and loss modulus (G”) were recorded for both experiments.

#### 2.3.5. Compression Properties

Sericin hydrogels and the sericin/i-PRF hydrogels were placed in cylindrical containers of 8 mm height and 12 mm diameter. The gels were then placed in a material universal tester (Instron 5848 Micro Tester, USA), and the analysis was conducted at test parameters set to a 1 mm/min displacement velocity at 25°C. The gels were compressed to 40% of the initial height, and the pressure (Pa) was plotted as a function of strain.

#### 2.3.6. Fourier Transform Infrared (FTIR) Spectroscopy Analysis

The test samples were dried and ground to a powder and then analyzed using an FTIR system (Nicolet iS20, Thermo Scientific) in the 400–4000 cm^−1^ region.

#### 2.3.7. *In Vitro* Growth Factor Release Test

GF release from i-PRF and sericin/i-PRF hydrogel was assessed by ELISA to determine the releasability of the gels. Briefly, 0.5 mL of i-PRF and 1 mL of sericin/i-PRF hydrogel (containing 0.5 mL of i-PRF) were added to a 6-well plate followed by 2 mL of high sugar-Dulbecco's Modified Eagle medium (Gibco, America) at 37°C. The media were collected and stored at -80°C on days 1, 3, 7, 11, 14, and 21 and replaced with equal volumes of fresh culture medium. The concentrations of PDGF, TGF-*β*1, VEGF, and EGF, in the collected media, were quantified using the ELISA kit according to the manufacturer's instructions.

### 2.4. Biocompatibility and Bioactivity of the Gels

#### 2.4.1. Cytotoxicity Assay

The cytotoxicity of the gels was evaluated by MTT assay [24]. Briefly, L929s were added to a 96-well plate (1 × 10^4^ cells/well) containing 100 *μ*L of cell culture medium (Gibco, America) with 10% fetal bovine serum (FBS). After 24 h of incubation under standard conditions, the wells were replaced with fresh culture medium containing a predetermined concentration of the sericin hydrogel, i-PRF, or sericin/i-PRF hydrogel. The culture medium was removed after 24 h of incubation, and 100 *μ*L of MTT agent (0.5 mg/mL) was added to each well. After 4 h of incubation, the MTT agent was removed, and 100 *μ*L of dimethyl sulfoxide was added to each well and shaken to dissolve the crystals. Lastly, the optical density (OD) of each well was measured at 490 nm using a Multiskan enzyme labeler (Thermo Fisher, China), and the cell viability of each sample was analyzed using the following formula:

(2) $$\begin{array}{c} \text{Cell viability}\left(\%\right)={\left[\text{OD}\right]}_{\text{test}}/{\left[\text{OD}\right]}_{\text{control}}\times 100\% \end{array}$$

where [OD]_test_ and [OD]_control_ are the ODs of the wells with hydrogel (test) and wells without hydrogel (control), respectively.

#### 2.4.2. Cell Proliferation Assay

The cell proliferation-ability of the gels was evaluated using a cell counting kit- (CCK-) 8 (Solarbio, China). Each well was inoculated with 2 × 10^3^ L929s in a 100 *μ*L cell culture plate containing 10% FBS. After 24 h of incubation under standard conditions, the culture medium was replaced with fresh culture medium containing 0.5 *μ*L sericin hydrogel, 0.5 *μ*L i-PRF, or 1 *μ*L sericin/i-PRF hydrogel. On days 1, 2, 3, and 4, 10 *μ*L of CCK-8 reagent was added to each well, and after 2 h of incubation, the OD of each well was measured at 450 nm using the Multiskan enzyme calibrator.

#### 2.4.3. *In Vivo* Biodegradation

Nude mice were used to evaluate the *in vivo* degradability of the gels to determine their biocompatibility. A total of 12 mice were first anesthetized with an intraperitoneal injection of 0.3% sodium pentobarbital (50 mg/kg), and two 1 cm incisions were made on each side of the back. Thereafter, the skin was incised, and the subcutaneous tissue was separated, after which 0.5 mL of the gel was subcutaneously injected into the mice. The gel and the surrounding tissues were collected at 6, 12, 48, 96, and 120 h postinjection and weighed. The degradation rate was measured by calculating the weight change of the gel remaining in the subcutaneous area. Experiments were carried out in accordance with approved Ethics Review Form for Animal Welfare (No.: 202208-201).

### 2.5. Application of the Sericin/i-PRF Hydrogel to a Full-Thickness Skin Defect Wound in a DM Nude Mice

#### 2.5.1. Type 2 DM Mouse Model

For this study, a total of 48 male nude mice (4-week-old; 15–20 g) were obtained and housed at 60% ± 5% relative humidity and 22 ± 2°C under a 12 h light/dark cycle. The mice had ad libitum access to food (a diet of regular food for immunodeficient animals) and homemade distilled water. The animal house was strictly disinfected once a day, and the animal bedding was changed every 3 days. The mice were kept in individually ventilated cages (IVC) for a week to observe their growth. After one week of acclimatization, the mice were maintained on a Co60 high-fat diet (HFD), and their body weight was recorded weekly. After 4 weeks, the mice were injected with streptozocin (STZ) and subjected to 12 h of fasting without water. The mice were then intraperitoneally injected with 1% STZ at 50 mg/kg of body weight. After one week, the blood glucose values were measured by tail vein blood collection, and the full-thickness skin defect wound was performed.

#### 2.5.2. Full-Thickness Skin Defect Wound Model

Nude mice were anesthesized as described previously, after which a circular 6 mm diameter full-thickness skin defect wound (to the myotendinous membrane) was made on their backs. A silicone ring (inner diameter: 8 mm) was sutured externally with 5-0 sutures to the skin to prevent skin contraction.

#### 2.5.3. Wound Healing Analysis

The diabetic wound mouse models (*n* = 48) were divided into normal saline (NS), sericin hydrogel, i-PRF, and sericin/i-PRF hydrogel groups and injected with NS (control) or the corresponding gels at the wound sites using a 1 mL syringe and left for approximately 15 min. The wounds were protected by sterilized transparent dressings and the dressing was changed every 2 days to assess the healing process. The wounds were photographed with a digital camera on days 0, 3, 7, 10, and 14. The wound areas were measured by two independent operators using image analysis software (ImageJ 1.53 k) [25], and the average values were calculated. The wound healing rate was calculated for each day using the following formula:

(3) $$\begin{array}{c} \text{Wound healing rate}=\left(A_{0}-A_{t}\right)/A_{0}\times 100\% \end{array}$$

where *A*_0_ is the initial wound area on day 0, while *A*_*t*_ is the wound area on the specified day.

#### 2.5.4. Histopathological Analysis

Six nude mice from each group were euthanized on days 7 and 14 after anesthesia, and skin tissue samples (2 mm along the wound edge) were obtained. The tissues were fixed in 4% paraformaldehyde, rinsed in a fixative after 24 h, gradient dehydrated, wax dipped, paraffin-embedded, and sectioned (5 *μ*m). The sections were then stained with hematoxylin and eosin (HE) and Masson staining, and histological patterns and collagen deposition were observed under a light microscope.

#### 2.5.5. Immunofluorescence (IF) Staining

Immunofluorescence staining for platelet-endothelial cell adhesion molecules (CD31) was performed on dewaxed and rehydrated tissue sections. Briefly, the sections were placed in an antigen-repair cassette containing EDTA antigen repair buffer (pH 8.0) and placed in a microwave oven. The sections were dried slightly, and circles were drawn around the tissue with a histochemical pen. After PBS dried, BSA was added dropwise, and the sections were enclosed in a box for 30 min. Subsequently, the sections were incubated with secondary antibodies for 50 min at 25°C in the dark. Lastly, the nuclei were stained with DAPI to quench the autofluorescence of the sections. The sections were observed and photographed under an inverted fluorescent microscope (Zeiss AXIO-VERT).

#### 2.5.6. Immunohistochemistry (IHC) Staining

The tissue sections were IHC stained for VEGF, TGF-*β*1, IL-6, and TNF-*α*. Thereafter, the sections were dewaxed and rehydrated and placed in an antigen-repair cassette containing citrate antigen repair buffer (pH 6.0) and placed in a microwave oven. For blocking endogenous peroxidase, the sections were placed in a 3% hydrogen peroxide solution and incubated for 25 min at 25°C in the dark. The slides were placed in PBS (pH 7.4) on a decolorization shaker and washed thrice for 5 min each. The sections were first treated with 3% BSA, to avoid nonspecific binding, and then incubated overnight with the primary antibodies at 4°C. After which, they were incubated with HRP-labelled secondary antibodies. DAB was added dropwise, and the staining time was controlled under the microscope. IHC-positive cells were stained brownish yellow under the light microscope, and the percentage of positive area for each index was analyzed using the ImageJ software. Sections from 4 animals were taken in each group, and 3 random fields of view were taken from each section.

This study was approved by the Animal Ethics Committee and adhered to the relevant guidelines.

### 2.6. Statistical Analysis

Data are expressed as mean ± standard deviation. Statistical comparisons were performed using one-way ANOVA, and ^∗^*p* < 0.05 and ^∗∗^*p* < 0.01 were defined as statistically significant.

## 3. Results and Discussion

### 3.1. Gel Characterization

#### 3.1.1. Formation and Morphology of the Sericin/i-PRF Hydrogel

**Figure 1:**
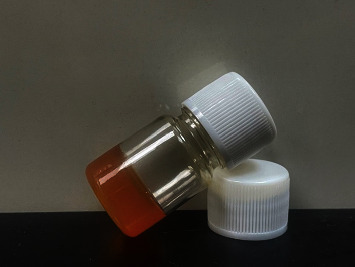
Sericin/i-PRF hydrogel.

The gels underwent liquid-to-solid transformation within 4 min at 25°C (Figure 1).

#### 3.1.2. Microstructure

**Figure 2:**
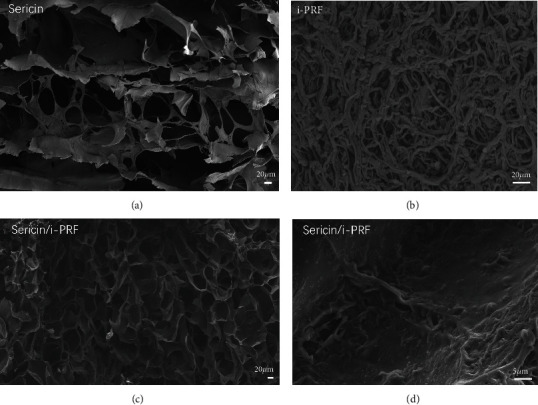
(a–d) Ultrastructures of sericin hydrogel (a), i-PRF (b), sericin/i-PRF hydrogel (c), and sericin/i-PRF hydrogel pore wall (d).

Dressings with a porous network structure facilitate the transfer of nutrients and oxygen to the wound site, absorb wound secretions, and maintain a moist wound environment, which facilitates wet wound healing [26]. SEM analysis showed that both sericin and sericin/i-PRF hydrogels have a typical 3D structure with interconnected pores. However, the sericin/i-PRF hydrogel had small and homogeneous pore sizes and a dense network. Furthermore, at higher magnifications, we observed a wall fibrin network in i-PRF, which demonstrates the interintegration of i-PRF and sericin hydrogel. Moreover, the fibrin bundles in the i-PRF were intertwined and connected, showing a complex meshwork fused into sheets (Figure 2).

#### 3.1.3. Gelation Time

**Figure 3:**
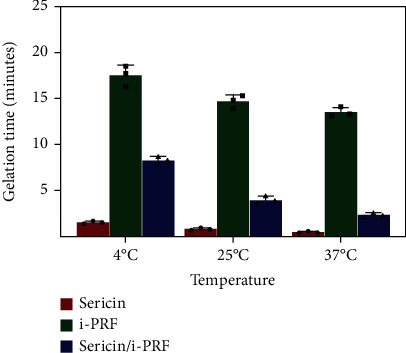
Gelation times of the sericin hydrogel, i-PRF, and sericin/i-PRF hydrogel (*n* = 3).

Sericin hydrogel and i-PRF were mixed, and the crosslinking agent was added immediately. After the activation of fibronectin and crosslinking of sericin, the sericin hydrogel and i-PRF fused to form an interconnected colloidal network (sericin/i-PRF hydrogel) [27]. We observed that the gelation time decreased with an increase in temperature, resulting in faster gel formation. For instance, an increase in temperature from 4°C to 37°C decreased the gelation time of the sericin/i-PRF hydrogel from approximately 8 min to 3 min (Figure 3). Furthermore, the rapid injectability of the sericin/i-PRF hydrogel allowed it to adapt to a variety of irregular wounds and be fully functional.

#### 3.1.4. Swelling Ratio

**Figure 4:**
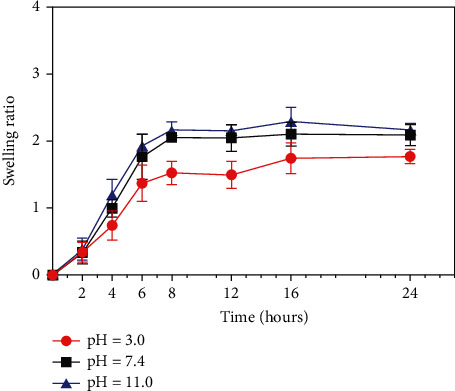
Swelling ratios of the sericin/i-PRF hydrogel at pH 3.0, 7.4, and 11.0 (*n* = 3).

The degree of expansion of the hydrogel is associated with the pore size of the material and influences its mechanical strength. Swelling ratio analysis revealed that the sericin/i-PRF hydrogel had a high water absorption capacity, possibly due to its high porosity and hydrophilicity. Furthermore, its swelling ratio increased rapidly with time, reaching half of the maximum value within 4 h, after which it increased slowly. The highest swelling ratio (approximately 2) of the sericin/i-PRF hydrogel was appeared under alkaline or neutral conditions and was only slightly higher than that of acidic conditions (Figure 4). Altogether, these results indicate that the sericin/i-PRF hydrogel retains its water absorption ability even in complex diabetic wounds and can thus be used for wound healing.

#### 3.1.5. Rheological and Compressive Properties

**Figure 5:**
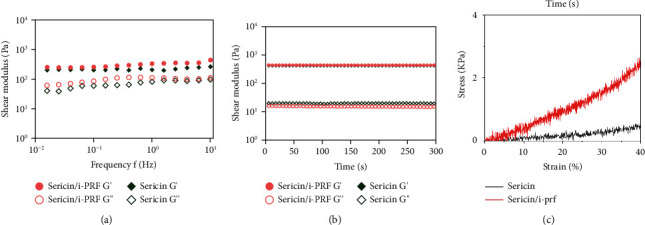
(a) Rheological properties of the sericin and sericin/i-PRF hydrogels between 0.1 and 10 Hz, (b) time-scanning experiment of the sericin and sericin/i-PRF hydrogels, and (c) compressive stress-strain curves of the sericin and sericin/i-PRF hydrogels.

In the frequency scan experiments, G' was higher than G” of the sericin/i-PRF hydrogel, at shear frequencies between 0.1 and 10 Hz, indicating that the hydrogel can maintain a stable 3D mesh structure and could exhibit good mechanical properties (Figure 5(a)). Whereas, in the time-scan experiments, G' was significantly higher than the G” of the sericin/i-PRF hydrogel, indicating that the hydrogel is an excellent elastomer. However, the G' of the sericin/i-PRF hydrogel was only slightly higher than that of the sericin hydrogel (Figure 5(b)). Additionally, compression analysis revealed that the sericin/i-PRF hydrogel can be compressed up to 40% without breaking, which is similar to the human skin (Figure 5(c)).

#### 3.1.6. FTIR

**Figure 6:**
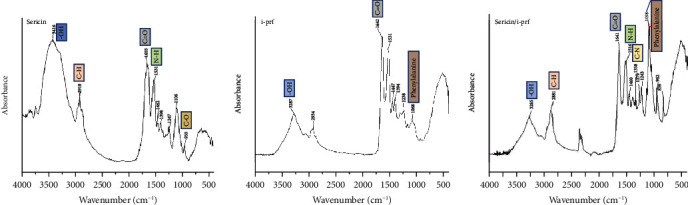
FTIR of the sericin hydrogel, i-PRF, and sericin/i-PRF hydrogel.

In the infrared (IR) spectrogram of the sericin hydrogel (Figure 6), the peak at 3416 cm^−1^ corresponds to a hydroxyl (OH) bond stretching vibration, peak at 2918 cm^−1^ corresponds to C–H bond stretching vibration in methylene, peak at 1655^−1^ cm corresponds to amide I with C=O bond stretching vibration, peak at 1531 cm^−1^ corresponds to amide II with N–H bond bending vibration, peaks at 1,462 and 1,398 cm^−1^ correspond to C–H bending vibrations in the hydrocarbon group, peak at 1,247 cm^−1^ corresponds to amide III with C–N stretching vibration, and peaks at 1,106 and 955 cm^−1^ correspond to C–O stretching vibrations. Since platelets are mainly composed of amino acids with aliphatic moieties, the IR spectrum of i-PRF revealed that the peak at 3287 cm^−1^ corresponds to OH stretching vibration, peak at 2934 cm^−1^ corresponds to C–H bond stretching vibration in methyl, peak at 1642 cm^−1^ corresponds to amide I with C=O bond stretching vibration, peak at 1531 cm^−1^ corresponds to amide II with N–H bond bending vibration, peaks at 1447 and 1394 cm^−1^ correspond to C–H bond bending vibration, peak at 1238 cm^−1^ corresponds to amide III with C–N bond stretching vibration, and peak at 1068 cm^−1^ corresponds to phenylalanine. Lastly, in the IR spectrum of the sericin/i-PRF hydrogel, the peak at 3285 cm^−1^ corresponds to OH stretching vibration, peak at 2881 cm^−1^ corresponds to C–H bond stretching vibration in methyl or methylene, the double peak near 2350 cm^−1^ corresponds to CO_2_ background absorption, peak at 1641 cm^−1^ corresponds to the amide I with C=O bond stretching vibration, and peak at 1516 cm^−1^ corresponds to C=O bond stretching vibration peak. Furthermore, the peak at 1516 cm^−1^ corresponds to amide II with N–H bond bending vibration, peaks at 1460 and 1338 cm^−1^ correspond to C–H bond bending vibration in the hydrocarbon group, peaks at 1279 and 1243 cm^−1^ correspond to amide III with C–N bond stretching vibration, and peak at 1098 cm^−1^ corresponds to C–O bond stretching vibration, which is characteristic of phenylalanine. Therefore, as seen in Figure 6, these results indicate the presence of the characteristic peaks of sericin and i-PRF in the sericin/i-PRF plot. Additionally, the peaks at 962 and 839 cm^−1^ correspond to C–H out-of-plane wobble vibrations, which indicate the integration of the sericin hydrogel with i-PRF.

#### 3.1.7. *In Vitro* Growth Factor Release Test

**Figure 7:**
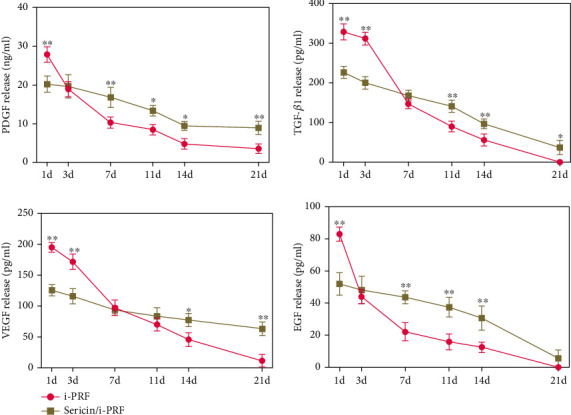
Release of the four major growth factors contained in i-PRF and sericin/i-PRF hydrogel (*n* = 3, ^∗^*p* < 0.05, ^∗∗^*p* < 0.01).

The release kinetics of the four GFs (PDGF, TGF-*β*1, VEGF, and EGF) were examined using ELISA to determine whether the hydrogel could prolong GF release. The results showed that the GF release profile of the sericin/i-PRF hydrogel was more uniform and slower than that of the i-PRF, with a longer release duration. These results suggest that the sericin/i-PRF hydrogel can provide a stable and prolonged supply of GFs and can be used for diabetic wound healing (Figure 7).

### 3.2. Biocompatibility and Bioactivity Tests

#### 3.2.1. Cytotoxicity Assay

**Figure 8:**
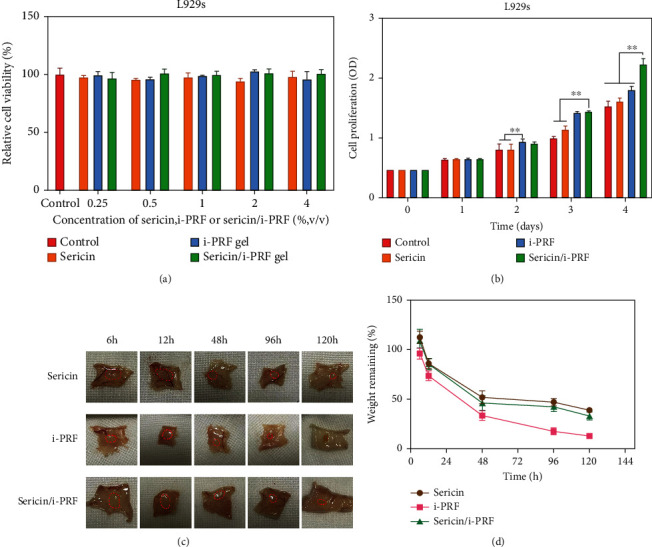
(a, b) *In vitro* cell viability assay (a) and cell proliferation assay (b) of the sericin hydrogel, i-PRF, and sericin/i-PRF hydrogel; (c) subcutaneous content of the sericin hydrogel, i-PRF, and sericin/i-PRF hydrogel after 6, 12, 48, 96, and 120 h postinjection (hydrogels are marked with red dotted lines); and (d) residual weights of the sericin hydrogel, i-PRF, and sericin/i-PRF hydrogel (*n* = 3, ^∗∗^*p* < 0.01).

The cytotoxicity assay (Figure 8(a)) revealed that the cell viability in the sericin, i-PRF, and sericin/i-PRF groups exceeded 90%, indicating that all these materials were noncytotoxic and safe for biomedical application.

#### 3.2.2. Cell Proliferation Assay

The cell proliferation assay revealed that the sericin/i-PRF hydrogel showed a more effective proliferation-promoting behavior than sericin hydrogel on day 4 (Figure 8(b)), which may be attributed to the fibroblast-proliferation abilities of the GFs in the i-PRF [28].

#### 3.2.3. *In Vivo* Biodegradation

Degradation is a fundamental characteristic of a biomaterial, which determines its biological application, safety, and application period [28]. The implantation sites of all the groups were visible at different time points with the surrounding tissue wrapping the hydrogel structure. Moreover, adverse reactions associated with gel injection, such as erythema or hyperplasia, were not observed in any of the groups. Among the three gels, i-PRF showed the most rapid volume shrinkage and weight loss postinjection, which may be due to the weak hydration of the fibrin network, causing the loss of its mechanical properties. Additionally, the sericin and sericin/i-PRF hydrogels had a residual weight of nearly 50% at 96 h postinjection, which may be attributed to the stability of sericin. Sericin in the sericin/i-PRF hydrogel protects the bioactive molecules in i-PRF from explosive release and prolongs their release behavior. Altogether, these results demonstrate the biocompatibility and superior structure of the sericin/i-PRF hydrogel (Figures 8(c) and 8(d)).

### 3.3. Sericin/i-PRF Hydrogel Promotes Wound Healing in DM Nude Mice

Diabetic wounds have been studied by generating full-thickness skin defect wounds in diabetic nude mice [29]. Delayed wound healing in diabetic patients is attributed to persistent inflammation at the wound site, vascular compromise, bacterial infection, and improper cytokine responses [30]. In this study, we first established an HFD/STZ-induced type 2 DM model in nude mice. Thereafter, we generated a full-thickness skin defect wound in the DM nude mice to simulate diabetic wounds. The diabetic wounds on the nude mice were then treated with NS, sericin hydrogel, i-PRF, and sericin/i-PRF hydrogel.

#### 3.3.1. Wound Healing Analysis

**Figure 9:**
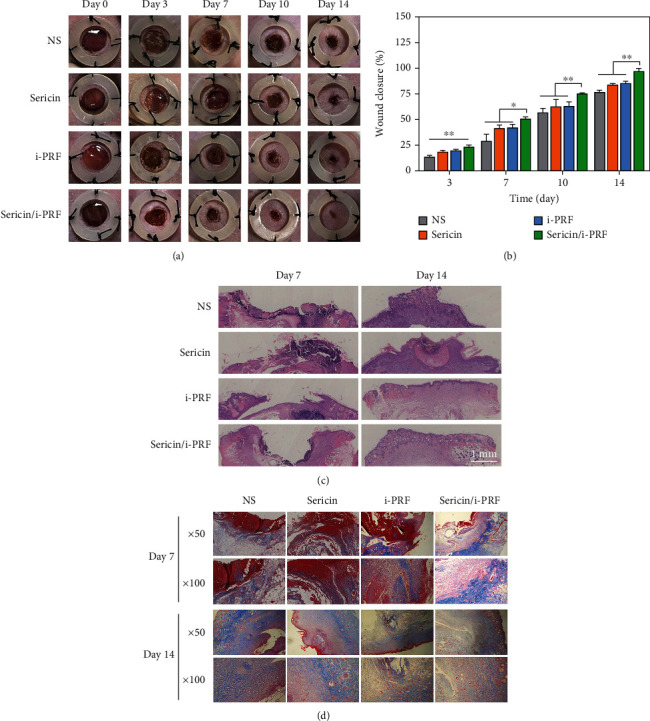
(a) Representative photos of the wounds in the NS, sericin hydrogel, i-PRF, and sericin/i-PRF hydrogel groups from day 0 to 14; (b) the wound healing rate of all the groups from day 3 to 14; (c, d) HE (c) and Masson (d) staining of the wound tissue sections of each group on days 7 and 14 (*n* = 4, ^∗^*p* < 0.05, ^∗∗^*p* < 0.01).

Although the wound area decreased over time in all the groups, wound closure was the fastest in the sericin/i-PRF hydrogel group. While the other groups showed some degree of inflammation on day 7 of the wound-healing process, the sericin/i-PRF hydrogel group showed only a mild inflammatory response. After 14 days of treatment, the wounds in the sericin/i-PRF hydrogel group were mostly healed, while the other groups showed significant residual wounds (Figure 9(a)). The wound healing rate was further quantified by calculating the wound repair area, and the results revealed that the healing rate of the sericin/i-PRF hydrogel group was significantly higher than that of the other groups. Furthermore, on day 14, the wound closure rate of the sericin/i-PRF hydrogel group was >95%, indicating that the reepithelialization process of the wound was largely completed (Figure 9(b)).

#### 3.3.2. Histopathological Analysis

HE staining was used to observe the morphological changes of the skin layers during the wound-healing process. On day 7, the sericin/i-PRF hydrogel group showed infiltration of some inflammatory cells and squamous epithelial cells, while the NS group showed inflammatory cell infiltration and had almost no epidermal and dermal tissue layers. Moreover, on day 14, the NS group showed the slowest reepithelialization, while the sericin/i-PRF hydrogel group was fully epithelialized, with a new uniform, dense, and continuous epidermis that approached normal skin thickness (Figure 9(c)). Masson staining was used to assess collagen deposition at the healing site to gain insight into the proliferative and reconstructive phases of the wound repair process. On day 7, collagen deposition was significantly higher in the sericin/i-PRF group compared to the other groups, while on day 14, collagen deposition increased but was loosely arranged and disorganized in the other groups, whereas the collagen fibers in the sericin/i-PRF hydrogel group were regularly arranged and closely resembled those in the normal skin (Figure 9(d)).

Granulation tissue is a new connective tissue in which a network of blood vessels initiates and supports skin regeneration. Granulation tissue also provides a matrix for the migration of keratin-forming cells, which activate the reepithelialization at the wound edges [31]. Disruption of the skin barrier provides an entry point for the infection of a wound, due to which epithelialization is impaired in almost all types of chronic wounds. Therefore, the rapid closure of wounds by accelerated migration and proliferation of epithelial cells plays a crucial role in wound healing [32]. Sericin/i-PRF hydrogel accelerates the wound repair process through faster reepithelialization and shorter wound closure time, which may help to reduce wound recurrence. Dermal repair is achieved through the migration and proliferation of fibroblasts, and the response of fibroblasts during wound healing determines the outcome of tissue repair [33]. Although collagen and extracellular matrix (ECM) deposition is necessary for effective wound closure [34], their disorderly arrangement can cause skin fibrosis and scar ormation [35]. The results of our study revealed that the sericin/i-PRF hydrogel allows the orderly deposition of collagen, which contributes to effective skin healing.

#### 3.3.3. IF and IHC Staining

**Figure 10:**
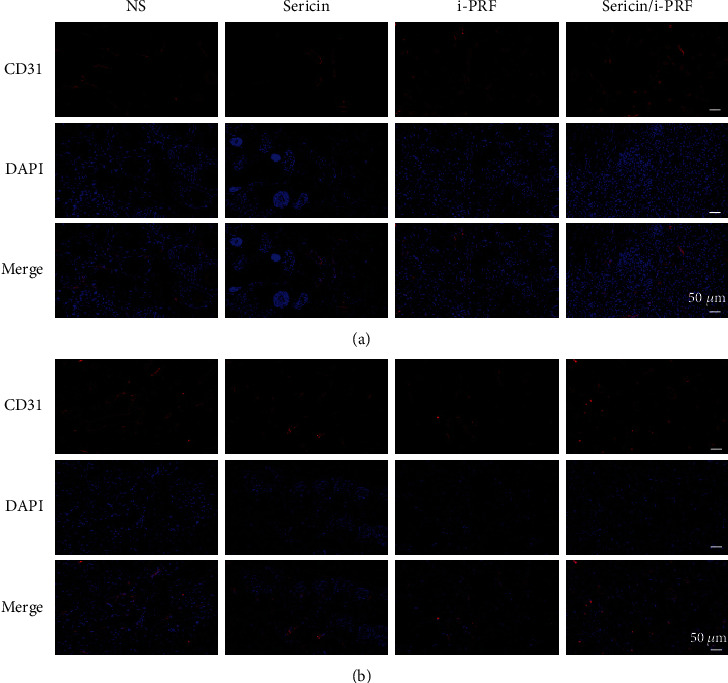
(a, b) IF staining of CD31 at wound margins in the tissue sections of the sericin hydrogel, i-PRF, and sericin/i-PRF hydrogel groups on days 7 (a) and 14 (b).

Hyperglycemia in DM may lead to vasoconstriction and inhibition of angiogenesis, which can hinder the wound-healing process by blocking the oxygen supply [36]. CD31 is a common marker of vascular endothelial cells, and the IF results showed that CD31 was significantly higher in the sericin/i-PRF group compared with the other groups, demonstrating that the sericin/i-PRF hydrogel can extensively and rapidly promote vascular regeneration (Figure 10).

**Figure 11:**
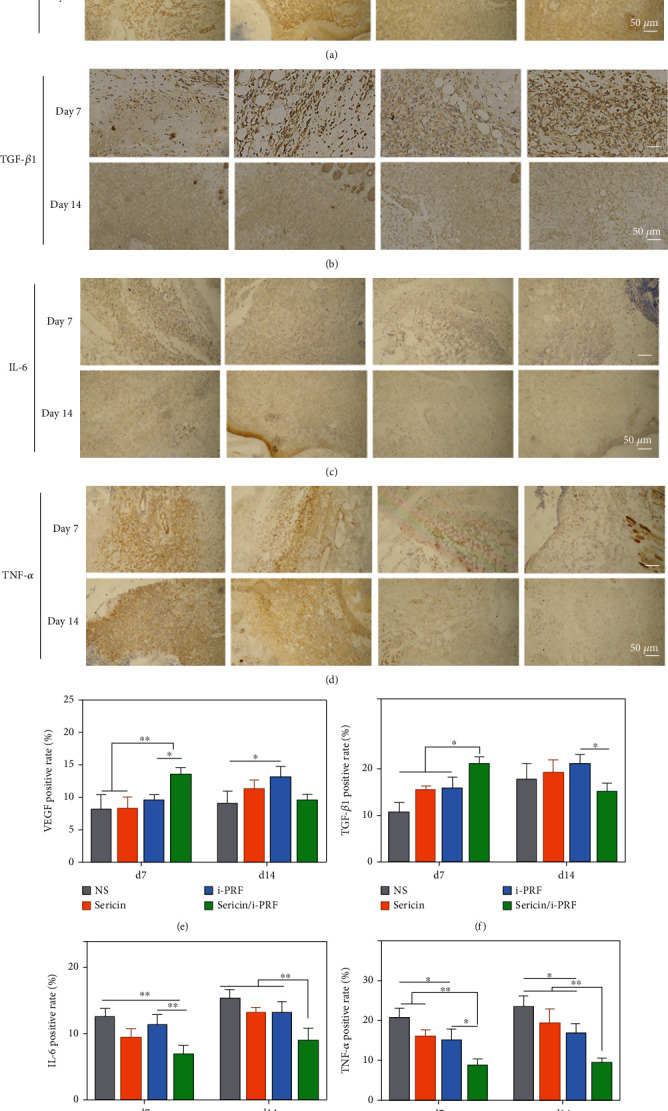
(a–d) IHC staining of VEGF (a), TGF-*β*1 (b), IL-6 (c), and TNF-*α* (d) in the tissue sections of the sericin hydrogel, i-PRF, and sericin/i-PRF hydrogel groups and (e–h) IHC positive rates of VEGF (e), TGF-*β*1 (f), IL-6 (g), and TNF-*α* (h) in the tissue sections of all the groups (*n* = 4, ^∗^*p* < 0.05, ^∗∗^*p* < 0.01).

VEGF, a key factor in promoting angiogenesis, facilitates the development of multiple stages of the angiogenic cascade, primarily the migration and proliferation of endothelial cells [37]. On day 7, VEGF secretion in the sericin/i-PRF hydrogel group was significantly higher than the other groups, indicating that the sericin/i-PRF hydrogel had proangiogenic effects on the wound-healing process. The results revealed that a physiological balance was achieved between angiogenic stimulators and inhibitors in the sericin/i-PRF hydrogel group, leading to vascular stabilization and accelerated wound healing (Figures 11(a) and 11(e)).

TGF-*β*1 is a multifunctional cytokine that upregulates collagen production and promotes vascular and granulation tissue formation. Additionally, TGF-*β*1 induces the conversion of monocytes to macrophages during the inflammatory phase of the wound-healing process [38]. Elevated TGF-*β*1 levels may be associated with favorable immunomodulatory effects, shortening the duration of the inflammatory phase in the early stages of wound healing. Furthermore, during the proliferative phase, TGF-*β*1-triggered fibroblasts can be converted into myofibroblasts. In addition, TGF-*β*1 can generate contractility at the ECM border, thereby promoting wound closure [39]. The IHC results showed that TGF-*β*1 expression was higher in the sericin/i-PRF hydrogel group on day 7, indicating that the sericin/i-PRF hydrogel contributes to TGF-*β*1 expression. The reason why the expression of TGF-*β*1 in sericin/i-PRF hydrogel group was lower than that in other groups on day 14 may be that the wounds in this group had basically healed, while the wounds in other groups were in the stage of proliferation. This may also be one of the reasons why sericin/i-PRF hydrogel can promote healing and thus reduce scars (Figures 11(b) and 11(f)). Furthermore, IHC staining for the wound proinflammatory factors, TNF-*α* and IL-6, showed that their expression levels were significantly lower in the sericin/i-PRF hydrogel group on days 7 and 14, indicating that the sericin/i-PRF hydrogel significantly downregulated the inflammation level at the wound site, thus playing a key role in the wound-healing process (Figures 11(c), 11(d), 11(g), and 11(h)).

These results suggest that the sericin/i-PRF hydrogel has a significantly stronger therapeutic effect in regulating relevant GFs and cytokines compared to the other agents, thus accelerating the wound-healing process.

## 4. Conclusion

In this study, we synthesized and characterized sericin/i-PRF hydrogel and tested its effectiveness in promoting diabetic wound healing. The sericin/i-PRF hydrogel, prepared via a simple one-step process by combining sericin hydrogel and i-PRF, was biocompatible and could adapt to irregular wounds. In addition, the sericin hydrogel could protect i-PRF in highly catabolic diabetic wounds, allowing for a prolonged release of GFs, thus increasing its *in vivo* therapeutic activity. Furthermore, the sericin/i-PRF hydrogel accelerated wound healing in DM nude mice by promoting angiogenesis, increasing collagen deposition, and reducing inflammatory responses. In conclusion, our results suggest that the sericin/i-PRF hydrogel has great potential in promoting diabetic wound healing.

## Data Availability

Data can be obtained from the author.
